# Antibacterial Agents for Composite Resin Restorative Materials: Current Knowledge and Future Prospects

**DOI:** 10.7759/cureus.57212

**Published:** 2024-03-29

**Authors:** Amnah A Algarni

**Affiliations:** 1 Restorative Dental Sciences Department, College of Dentistry, Taibah University, Madinah, SAU

**Keywords:** mdpb monomer, quaternary ammonium compounds, chitosan, chlorhexidine, composite resin, caries, antibacterial agent

## Abstract

Resin composites became the material of choice for direct restorations in anterior and posterior teeth. Despite the revolutionary improvement in the material, restoration failure is still a major drawback due to the material’s inherent negative properties, including a lack of antibacterial effects. Therefore, many attempts have been made to incorporate antibacterial agents into resin composite materials to improve their antimicrobial properties and prevent secondary caries formation. Multiple laboratory studies have been conducted using different antibacterial agents, such as quaternary ammonium compounds, methacryloyloxydodecylpyridinium bromide, magnesium oxide nanoparticles, chlorhexidine, and chitosan. This review provides a glance at the current status of these materials and the research directions needed in the future.

## Introduction and background

Composite resins became the most widely used materials for direct restorations due to the revolutionary improvement in mechanical, physical, and aesthetic properties [1]. However, the inherent disadvantages of these materials, including polymerization shrinkage stresses, moisture sensitivity, and lack of antibacterial properties, are still responsible for restoration failure [1]. It has also been found that composite resin materials are more susceptible to bacteria adherence and biofilm formation on their surface due to low surface energy [2]. In addition, the composition of conventional composite resin restorative materials does not show any bacterial inhibition effect. The concentration of monomers that leached out from composites is very low to allow antibacterial activity. The inorganic fillers are mainly inert silica fillers with no antibacterial properties as well [1]. The oral environment is dynamic where demineralization and remineralization occur simultaneously, and is affected by other several factors, such as salivary flow and the presence of fermentable carbohydrates [3]. The material’s negative properties with previously mentioned factors might eventually cause secondary caries and restoration failure [1]. The prevalence of secondary caries was found to be higher with resin composite restorations compared to other materials such as amalgam [4]. Thus, the failure rate of composite resin restorations is twice that of amalgam restorations, with secondary caries as the main cause of failure [5].

Several strategies have been introduced to enhance composite resin properties and reduce secondary caries initiation and progression. In an attempt to decrease bacteria colonization and plaque formation on the restoration surface, the resin composite material has been modified by the incorporation of antimicrobial agents. In modern dentistry, antibacterial dental resin composite restorative materials displayed great promise in resolving the long-standing issue of bacterial colonization and subsequent secondary caries formation in restored teeth [6]. Moreover, composite resin has also been modified by adding a combination of antimicrobial and remineralization agents [7,8]. This review aims to provide insight into available antibacterial agents that have been added to composite resin restorative materials to enhance the antimicrobial and anti-caries properties.

## Review

Antibacterial agents

**Figure 1:**
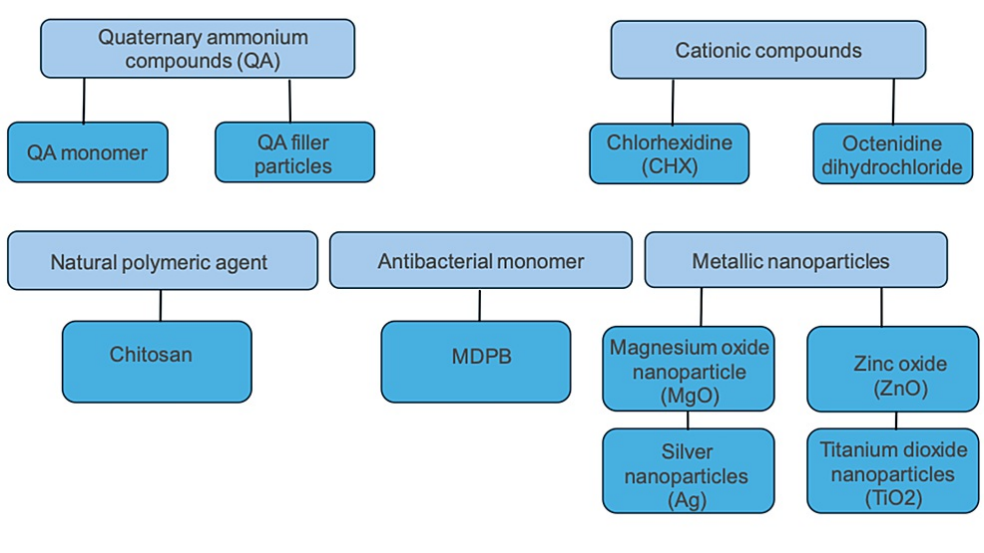
Review flowchart Flowchart summarizing the antibacterial agents being discussed in this review. MDPB: Methacryloyloxydodecylpyridinium bromide

Several antimicrobial agents have been tried and incorporated into composite materials. These agents can be classified according to their chemical composition into organic and inorganic agents. Organic agents include polymeric agents, quaternary ammonium compounds, and biguanides, while inorganic agents include metals and metal oxides. According to the method of incorporation into composite resins, antibacterial agents can also be classified as polymerizable agents, leachable agents, and antibacterial filler particles. Polymerizable antibacterial agents are incorporated into a composite resin matrix through copolymerization with resin monomers, providing antibacterial effects without the release of antibacterial components and ensuring long-term antibacterial protection. Methacryloyloxydodecylpyridinium bromide (MDPB) is an example of a polymerizable antibacterial agent. Leachable agents, such as quaternary ammonium compounds and chlorhexidine, are water-soluble compounds that can be released into the surrounding area of a composite resin restoration. Filler particles, including metal oxides or metal salts, such as magnesium oxide, display antibacterial action by releasing trace amounts of metal ions [6]. The antibacterial agents discussed in this review are summarized in Figure 1.

Quaternary Ammonium Compounds (QACs)

These compounds exhibit strong antibacterial agents and have been utilized for decades in food packaging and biomedical applications [9]. Likewise, they showed promising antibacterial effects when added to dental composites. Their mechanism of action is basically through releasing catalytic agents, which break down microbial membranes of bacteria and prevent their adherence [9]. Two primary methods exist for their incorporation into dental composite: either by being added as a filler particle component or they are polymerized as a QA monomer.

Compounds as a QA Monomer: Several in vitro studies have been conducted to test the effect of incorporation of QA monomers on the antibacterial and physical properties of dental composites. To maximize chain length and other properties of the compounds involved, QA dimethacrylate monomers were tested and used in dental resin systems containing bisphenol A-glycidyl methacrylate and trimethylene glycol dimethacrylate for antibacterial activity [10]. In vitro studies investigated copolymers based on triethylene glycol dimethacrylate and QA urethane-dimethacrylate analogs [11]. The tested copolymers exhibited acceptable physical properties as well as superior antibacterial properties. However, these experimental materials have not been tested clinically to be validated.

Compounds as QA Filler Particles: QA compounds can be either polymeric particles (e.g.QA polyethylenimine (QPEI)) or coupled with silica filler particles. These compounds have been thoroughly investigated throughin vitroand in vivostudies [12]. These compounds are more advantageous compared to QA monomers, as the latter tend to leach out the composite restorative materials resulting in deterioration in the physical properties of the material with time [6].

In laboratory studies, QPEI polymeric nanoparticles have been prepared and incorporated into bonding agents, and flowable and hybrid composite resin restorative materials at different concentrations ranging from 0.1 to 3 % (wt/wt). Most of these in vitro studies revealed a high antibacterial effect over time. An increase in solubility and a decrease in mechanical properties such as flexural strength were observed with concentrations above 1% [12, 13,14]. Few in-vivo studies have been conducted to test these materials intraorally with similar observations found regarding a high wide-spectrum antibacterial effect [15]. The QPEI caused cell death throughout the formed biofilm, and not just at the restorative material surface.

Regarding QA silica particles, several in vitro studies revealed a high antibacterial effect. Incorporation of QA silica particles into experimental composite materials at a concentration of 1.5% showed significant antibacterial activity with no negative impact on the mechanical properties of the material [14]. Moreover, QA silica particles are dispersed evenly throughout the dental material without being leached out, minimizing the risk of accumulation in the body. There are a limited number of commercially available restorative materials that contain QA silica particles. Infinix is a light-cured universal resin composite restorative material that has been introduced recently by Nobio (Infinix; Nobio, Israel), containing 1.5% QA silica particles. This material was tested in situ and showed a significant decrease in the metabolic activity of salivary bacteria when it encountered the material surface. This bacterial inhibition prevented salivary sugar- induced pH drop and maintained near neutral pH compared to other restorative materials, including amalgam and conventional resin composite [16]. Furthermore, the antibacterial activity was found to prevent tooth demineralization in enamel adjacent to a 38-μm gap for up to 4 weeks when compared to a conventional composite [16,17,18]. Clinical studies are needed to prove the effectiveness of these materials with time.

Methacryloyloxydodecylpyridinium Bromide (MDPB): The antibacterial monomer 12-methacryloyloxy dodecyl-pyridinium bromide is a combination between a methacryloyl group and quaternary ammonium [19]. At low concentrations (8 μg/ml), it displayed a bacteriostatic effect by interfering with bacterial lactate dehydrogenase enzyme activity and inhibiting acid production from *S. mutans*. At higher concentrations (1,000 μg/ml), MDPB has a bactericidal effect on planktonic and biofilm *S. mutans *[20]. 

MDPB has been incorporated into several restorative materials such as adhesive systems. Laboratory studies conducted on adhesive systems containing MDPB showed promising results regarding their antibacterial activity with no impact on tensile bond strength to dentin [21,22]. Clinical and in situ studies, however, were not congruent with the laboratory studies and showed no superior preventive effect against secondary caries formation between MDPB-containing and MDPB-free adhesives [23]. Moreover, no studies are available so far to investigate the incorporation of MDPB into restorative composite resin materials.

Chlorhexidine (CHX)

CHX is a biguanide cation with a broad-spectrum antibacterial activity against Gram-negative and Gram-positive bacteria. Its bacteriostatic mode of action relies on penetrating and damaging the bacterial cell, thus compromising the integrity of the cell. At higher concentrations, it exhibits a bactericidal effect by causing precipitations and cytoplasmic coagulation [24]. In the dental field, it has been used for centuries as an effective anti-bacterial mouthwash and varnish for the treatment and prevention of periodontal diseases and dental caries. In addition, CHX exhibits a matrix metalloproteinases (MMPs)-inhibition effect. MMPs are endogenous proteolytic enzymes within the dentin organic matrix. When activated, MMPs lead to the degradation of collagen fibers and the deterioration of the dentin-resin composite bond strength. CHX has been utilized in restorative procedures as a therapeutic primer, added to acid etch, and incorporated into adhesive agents [25]. A laboratory study showed that the application of 0.2% CHX solution as a therapeutic primer before the dentin bonding procedure would reduce nanoleakage [26]. CHX has also been incorporated into acid etching when bonding to eroded dentin and displayed a significant nanoleakage inhibition effect [27]. Despite the promising MMP inhibition effect of CHX provided by in vitro studies, not enough clinical evidence is available in the literature to provide strong clinical recommendations [28]. 

In addition to the application of CHX solution during bonding procedures, several trials have been performed in order to incorporate CHX into restorative materials to create anti-caries restorations. It has been incorporated into composite resins by using mesoporous silica nanoparticles to encapsulate and release CHX [29]. The resulting experimental composite showed controlled CHX release over time without compromising mechanical and surface properties. In another attempt, vesicle-templating technology was utilized to create core-shell CHX/ACP nanoparticles and incorporated into experimental composite resin restoration, following which their antibacterial and physical properties were tested. Acceptable mechanical, antimicrobial, and remineralization properties were displayed by the modified dental resin composite containing 5% CHX/ACP nanoparticles [30]. In addition, CHX was also incorporated into adhesive resin systems and showed promising results. Indeed, a meta-analysis study revealed that bonding agents containing 0.2% CHX enhanced bond strength with time. This could be explained not only by the antimicrobial effect, but also by being an MMP inhibitor exhibiting anti-proteolytic activity [31]. These observations might indicate the possibility of creating the perfect restorative material in the future.

Octenidine dihydrochloride, another cationic compound derived from pyridine that acts similarly to CHX, showed antibacterial and biofilm prevention effects. It has been incorporated into experimental composite resin and showed a promising anti-biofilm formation effect [32]. Further research is still needed as only a few in situ studies have investigated this material [32,33].

Chitosan

Chitosan is a chitin-derived polysaccharide that exhibits antimicrobial activity. It is considered a biocompatible and biodegradable natural polymeric antimicrobial agent that can be used in multiple biomedical applications and tissue regeneration [34]. Chitosan polymeric nanoparticles can be produced by polymer coagulation from solutions containing several concentrations of the material, which is subsequently crosslinked with sodium tripolyphosphate. The antimicrobial effect of chitosan nanoparticles is via bacterial cell membrane disruption. The large positively charged chitosan nanoparticles interact with the negatively charged bacterial cell wall, increasing the permeability of the bacterial cell membrane [35].

Chitosan nanoparticles have been utilized for targeted drug delivery for several therapeutic uses in dentistry, including treatment of periodontitis and dentin-pulp regeneration, as well as incorporation into dental adhesive systems and resin composites [36]. Controversial results have been reported regarding the antibacterial effect of chitosan addition into composite resin and the impact of this addition on the mechanical properties of the material. A previous report [37] added chitosan into methacrylate, which was successfully incorporated into a total-etch bonding system with no adverse influence observed on bond strength and physical properties. In another laboratory study, the incorporation of chitosan-composite resin and adhesives showed a significant reduction in *S. mutans* biofilm formation. This increase in the antibacterial effect of the experimental composite led to a reduction in mechanical properties such as surface hardness and flexural strength [38]. Lia et al. reported that the incorporation of 2% chitosan/fluoride microparticles into the bisphenol A-glycidyl methacrylate (Bis-GMA) resin matrix resulted in a significant reduction in bacterial growth to 10% without compromising the mechanical properties of the material [39]. Likewise, 0.25% of chitosan particles incorporated into composite or dentin bonding agents exhibited antibacterial action without impacting the bond strength when tested in class II restorations in vitro [40]. On the other hand, improved microhardness was observed when different concentrations of chitosan were added to the resin composite (0.5-1%); no significant bacterial inhibition effect was compared to the chitosan-free control [41]. Overall, the inconclusive results obtained by the in vitro studies necessitate the need for further investigations with more standardized methods to optimize the concentration of chitosan particles and properties of the resulting composite material. 

Metallic Nanoparticles

Magnesium oxide nanoparticle (nMgO): Magnesium oxide nanoparticles are biocompatible substances with promising antimicrobial properties [42]. They have been utilized in cancer treatment for tumor inhibition, bone regeneration, and stomach pain relief [43]. MgO nanoparticles also have been found to exhibit antimicrobial properties against oral pathogens. Their mode of action relies on the release of magnesium ions causing the disruption of the bacterial cell membrane and a reduction in bacterial growth [44]. Therefore, it has been incorporated as an antibacterial filler into resin composites, dental cement, and glass-ionomer cement, to prevent secondary caries formation [45,46]. At low concentrations (2-7.5% by weight), this addition was found to greatly enhance anti-biofilm properties without compromising their mechanical, physicochemical, or biocompatibility properties [47,48]. Furthermore, it's possible that a minimum quantity of MgO nanoparticles improved resin composite polymerization and enhanced the depth of curing, as well as decelerating the aging process of the material [47,48]. This could be a useful tool in the development of antibacterial resin composites to prevent secondary caries formation.

Zein protein is a polymer derived from corn and characterized by being anti-film-forming, biodegradable, and biocompatible. In combination with MgO nanoparticles, it enhances their properties and makes the nanoparticles suitable for drug delivery and therapeutic agents for different biomedical applications. Zein-coated MgO nanoparticles can be prepared via several techniques, including covalent bonding or adsorption. Zein-coated MgO displayed promising antibacterial properties against *Streptococcus mutans*, *Enterococcus faecalis*, *Staphylococcus aureus*, and *Candida albicans* (as oral fungus) [49]. Besides, when Zein-coated MgO nanoparticles were incorporated into resin-modified glass ionomer cements and resin cements, they showed a prominent antibacterial effect [45].

MgO has also been accompanied with bioactive glass (BAG) and incorporated into composite resin. MgO-BAG is suggested to exhibit an antimicrobial effect from inorganic MgO, as well as a remineralization effect from organic BAG. When 2.5 wt% MgO and 12.5 wt% BAG were simultaneously incorporated into a composite resin, it displayed higher antimicrobial activity than the ones containing only MgO or BAG. This combination did not affect the physical, mechanical, and chemical properties of the modified composite resin material [50]. Overall, MgO nanoparticles were promising antibacterial fillers for dental composites; however, limited research is available to provide strong evidence for clinical use. This emphasizes the need for further research in this area.

Zinc oxide (ZnO): ZnO nanoparticles are considered insoluble and colorless particles that provides long-lasting antibacterial properties. Thus, they would be suitable as antibacterial filler nanoparticles in a direct resin composite restorative material. There was a significant increase in the antibacterial effect of composite resins that contain 1-2 wt % ZnO nanoparticles compared to controls [51]. The highest antibacterial effect has been observed with the addition of 5% ZnO nanoparticles by reducing lactic acid production by cariogenic bacteria [52]. However, some studies suggested that ZnO nanoparticles are mainly effective on a single species rather than multispecies biofilm.

Within low concentrations (1-2%), ZnO nanoparticles provide an antimicrobial effect without affecting mechanical properties, bond strength, or degree of conversion of the dental composite. Controversial results were observed for higher concentrations (5%), as one study reported a significant reduction in curing depth and degree of conversion of the material. However, there were other studies no negative impact was reported [52,53]. A finite study conducted by Yazdani et al. supported that the addition of 5% ZnO particles into resin composite provides the best thermo-mechanical behavior [54].

Silver nanoparticles: Silver nanoparticles (AgNPs) are used as antibacterial agent for several medical and dental uses. Its antibacterial action relies on multiple methods including the disruption of the cytoplasmic membrane and cell wall, ribosome denaturation and inhibition of protein synthesis, as well as interruption of ATP formation [55].

Yassaei et al. suggested that 1% is the maximum concentration of AgNPs to be incorporated into resin composite material to provide the best antibacterial effect without affecting the materials’ properties. Increasing AgNP concentrations might increase the risk of toxicity and deterioration in the aesthetic properties of the material [56]. A more recent study where resin composite was modified by adding 1-1.5% Ag nanoparticles extracted from a plant (*Equisetum sylvaticum*) displayed significant antibacterial activity against *Streptococcus mutans* with no effect on the materials’ surface hardness [57].

Several attempts have been made to use a combination of Ag with other antibacterial agents for superior properties. Arif et al. investigated the incorporation of Ciprofloxacin with Ag nanoparticles into resin composite material. The modified material was biocompatible and displayed significant antibacterial activity as well as improved compressive strength compared to the control group [58]. Moreover, an orthodontic composite resin loaded with a combination of ZnO and Ag nanoparticles was also explored and found to significantly enhance, both, the antibacterial properties and the bond strength to tooth structure [59].

Titanium dioxide nanoparticles (TiO_2_): TiO_2_ nanoparticles are considered as photocatalytic antibacterial agent fillers that can be activated by absorbing UV radiation and releasing reactive oxygen species [60]. Recently there have been attempts to increase light absorption of TiO_2_ nanoparticles by creating doped nanoparticles using different elements such as nitrogen without affecting the materials’ antimicrobial properties. In a study investigating composites used for orthodontic retainers, TiO_2_ nanoparticles containing resin composite showed significantly higher bacterial inhibition compared to conventional composite without any impact on bond strength [61]. The combination of TiO_2_ and Ag nanoparticles also showed substantial improvement in the mechanical properties of dental composite in addition to the antibacterial effect. Likewise, the incorporation of 1.5% nitrogen and fluorine-doped TiO_2_ enhanced the antibacterial effect in addition to mechanical properties including flexural strength and flexural modulus of flowable resin composites [62]. Despite several studies supporting the use of TiO_2_ nanoparticles as an antibacterial agent, further research is still needed to determine the exact composition and concentration of the nanoparticles to be incorporated into resin composite materials.

Research gap and future directions

There are several adhesive systems with antibacterial properties available in the market. However, only one restorative composite resin product exists in the market for clinical use containing QA silica particles (Infinix; Nobio, Israel). No longitudinal clinical trials have been conducted and published on this product to prove its clinical performance in comparison to conventional and bulk-fill resin composite materials.

In-vitro studies are needed to explore the impact of antibacterial agents’ incorporation on the depth of curing and degree of conversion, microleakage, and bond strength to enamel and dentin, as well as compatibility with different adhesive systems. Color stability, discoloration, and wear resistance need to be tested immediately and after aging periods. Researchers are also encouraged to conduct in situ studies to investigate the impact of human saliva and oral environments on the materials’ antibacterial, mechanical, and physical properties. Research must continue to find the perfect composition and ratios of the antibacterial agents that provide the best antibacterial effects without affecting the mechanical and physical properties of the composite material. Subsequently, translational research should be conducted to bridge the gap between laboratory studies and clinical practice.

## Conclusions

The knowledge available in the literature regarding antibacterial composites are mainly at the level of basic research. Laboratory studies and basic research provided promising results regarding the performance of antibacterial composite resin restorative materials. Translational research is needed to move forward with the production of such materials for clinical use. The production of such materials will improve caries control and management, as well as improve the longevity of resin composite restorative materials.
